# Global report on preterm birth and stillbirth (4 of 7): delivery of interventions

**DOI:** 10.1186/1471-2393-10-S1-S4

**Published:** 2010-02-23

**Authors:** Cesar G Victora, Craig E Rubens, the GAPPS Review Group

**Affiliations:** 1 Universidade Federal de Pelotas, Pelotas 96001-970, Brazil; 2 Global Alliance to Prevent Prematurity and Stillbirth, an initiative of Seattle Children's, Seattle, Washington, USA; 3 Department of Pediatrics at University of Washington School of Medicine, Seattle, Washington, USA

## Abstract

**Background:**

The efficacious interventions identified in the previous article of this report will fail unless they are delivered at high and equitable coverage. This article discusses critical delivery constraints and strategies.

**Barriers to scaling up interventions:**

Achieving universal coverage entails addressing major barriers at many levels. An overarching constraint is the lack of political will, resulting from the dearth of preterm birth and stillbirth data and the lack of visibility. Other barriers exist at the household and community levels, such as insufficient demand for interventions or sociocultural barriers; at the health services level, such as a lack of resources and trained healthcare providers; and at the health sector policy and management level, such as poorly functioning, centralized systems. Additional constraints involve weak governance and accountability, political instability, and challenges in the physical environment.

**Strategies and examples:**

Scaling up maternal, newborn and child health interventions requires strengthening health systems, but there is also a role for focused, targeted interventions. Choosing a strategy involves identifying appropriate channels for reaching high coverage, which depends on many factors such as access to and attendance at healthcare facilities. Delivery channels vary, and may include facility- and community-based healthcare providers, mass media campaigns, and community-based approaches and marketing strategies. Issues related to scaling up are discussed in the context of four interventions that may be given to mothers at different stages throughout pregnancy or to newborns: (1) detection and treatment of syphilis; (2) emergency Cesarean section; (3) newborn resuscitation; and (4) kangaroo mother care. Systematic reviews of the literature and large-scale implementation studies are analyzed for each intervention.

**Conclusion:**

Equitable and successful scale-up of preterm birth and stillbirth interventions will require addressing multiple barriers, and utilizing multiple delivery approaches and channels. Another important need is developing strategies to discontinue ineffective or harmful interventions. Preterm birth and stillbirth interventions must also be placed in the broader maternal, newborn and child health context to identify and prioritize those that will help improve several outcomes at the same time. The next article discusses advocacy challenges and opportunities.

## Introduction

Once cost-effective interventions are identified and prioritized, appropriate strategies must be used to scale-up delivery to reach high and equitable population coverage and reduce the global burden of disease (see article 3 for a discussion on existing preterm birth and stillbirth interventions [1]). Appropriate delivery strategies are those tailored to match the unique needs and capacities of specific regions or populations within each country.

This article begins with a general discussion of barriers and approaches to scaling up interventions. While the focus is on low- and middle-income countries (LMICs), some of the discussion will also be applicable to high-income countries. This is followed by a discussion of choosing cost-effective interventions. Four specific interventions are then used as examples. The article concludes with a discussion of scaling interventions in the broader maternal, fetal, newborn and child health context.

## Barriers to scaling up delivery of interventions

Table 1 summarizes barriers to achieving universal coverage of preterm birth and stillbirth interventions. This table is an adapted typology of the main constraints to scaling up child survival interventions in LMICs [2,3]. Barriers exist at multiple levels, from households to health services, and throughout different political and physical environments. Depending on the intervention being implemented, different types of constraints may operate.

**Table 1:** Main Constraints to Scaling Up Preterm Birth and Stillbirth Interventions in LMICs

Level of Constraint	Types of Constraints
Community and	• Insufficient demand for effective and available interventions
Household Level	• Barriers to use of effective interventions (e.g., physical, financial, and sociocultural)
Health Services Delivery Level	• Shortage and distribution of appropriately qualified healthcare providers
	• Weak technical guidance, program management and supervision
	• Inadequate pharmaceutical products and medical supplies
	• Lack of equipment and infrastructure
	• Poor accessibility of health services
Health Sector Policy and	• Weak and overly centralized systems for planning and management
Strategic Management Level	• Lack of competent district health management teams
	• Weak drug policies and supply system
	• Inadequate regulation of pharmaceutical and private sectors
	• Improper industry practices
	• Poorly functioning health information systems
	• Lack of intersectoral action and partnership for health between government, industry and civil society
	• Weak incentives to use inputs (e.g., medicines and laboratory tests) efficiently and respond to user needs and preferences
	• Difficulty in scaling up successful interventions to the national level
	• Monitoring and evaluating programs
	• Reliance on donor funding that reduces flexibility and ownership
	• Donor practices that damage country policies
Public Policies Cutting	• Government bureaucracy (civil service rules and remuneration, centralized management system, civil service reforms)
Across Sectors	• Poor availability of communication and transport infrastructure
Visibility of the Problem	• Lack of data on the magnitude of preterm birth and stillbirth
	- broad measurement issues (e.g., sources of data)
	- need for better operational definition of stillbirth
	- need to distinguish antepartum and intrapartum deaths
	- need for better measurement of preterm birth (i.e., not based on birth weight)
	- better identification of preterm birth and low birth weight
	• Lack of political visibility of the problem of preterm birth and stillbirth at country and international levels
Environmental and	• Governance and overall policy framework
Contextual Characteristics	- corruption, weak government, weak rule of law and enforceability of contracts
	- political instability and insecurity
	- weak ministry of health
	- low priority attached to social sectors
	- weak structures for public-sector accountability
	- lack of free press
	• Physical environment
	- climatic and geographic predisposition to disease
	- physical environment unfavorable to service delivery

Source: Hanson, K., et al., Victora, C.G., et al. [2,3]

Although many of the most significant barriers to delivery of effective interventions reside within the health systems and Ministries of Health, the solutions to these barriers often reside in more powerful areas of government such as Ministries of Finance and Planning, as well as in Ministries of Foreign Affairs which deal with foreign aid and international financial organizations. This is essential for addressing a key constraint which limits the scaling up of interventions— namely, affordability. As Cleary and Mclntyre note, "even if a conclusion is reached that a particular strategy is deemed cost-beneficial… it does not follow that it is necessarily affordable, particularly given the extremely constrained health-care resources in many African countries [4]."

An overarching constraint is the lack of political interest in preterm birth and stillbirth. This is largely attributed to low visibility associated with the inherent difficulties in measuring these outcomes. Addressing these data gaps is an essential step in highlighting the importance of these problems, as discussed in the article 1 on data [5], article 2 on etiologies [6], and article 5 on ethics [7].

## Approaches for scaling up

Authors reviewing strategies for preventing maternal [8,9] and neonatal [10,11] deaths emphasize the need to build functional health systems. This includes ensuring geographic and financial access to poor populations; training, deploying, and retaining health workers; and guaranteeing supplies of commodities and drugs. The recent revival of the "Health for All" approach adopted 30 years ago at the Alma-Ata Conference supports the need to strengthen health systems [12].

A distinct approach was adopted by a 2007 review [13] to assess the potential of scaling up maternal, fetal, newborn and child health interventions. The authors reviewed 43 promising health interventions portrayed as proven effective in reducing neonatal, child, and maternal morbidity and mortality. They excluded 22 interventions that required extensive behavioral changes, laboratory testing or advanced clinical skills. A "best-bets" analysis was done of the remaining 21 interventions. Two of these 21 best-bet interventions have a potential impact on preterm rates: insecticide-treated materials (mainly bed nets) for malaria prevention and intermittent presumptive treatment for malaria in pregnancy. A third intervention affects the survival of preterm infants— corticosteroids given during preterm labor. Six criteria were used to select promising interventions:

1. simplicity (no need for a sophisticated delivery system);

2. compatibility (with existing treatment and prevention norms of providers and clients);

3. public health impact (in terms of morbidity and mortality);

4. observability (ease of monitoring and evaluating impact);

5. cost (user and provider costs); and

6. relative advantage (compared to other interventions addressing the same problem).

Although it is reassuring that three interventions related to preterm birth were included, the criteria employed by the authors favored stand-alone, vertically-delivered interventions as opposed to the horizontal approach of strengthening health systems to deliver packaged interventions. However, even typically "vertical" approaches such as vaccination campaigns, for example, require trained health workers, supervision, an information system, consumables and equipment for the cold chain.

Recently, there is increasing interest in the concept of the "continuum of care," encompassing reproductive, antenatal, delivery, postnatal/neonatal, and child care [14]. In addition to its lifecycle or temporal dimension, the continuum also refers to the different levels or settings where care must be provided—households and communities, outreach and outpatient services, and inpatient care. This strategy favors the horizontal delivery of packages through cost-effective interventions by strengthening health systems, in contrast to the vertical approach promoted by the Gillespie review of scalable, stand-alone interventions with limited emphasis on building a functional health system [13].

The "vertical vs. horizontal" debate has been denounced for being somewhat artificial. A combination of both is required for scaling up effective interventions namely the "diagonal" approach [3,15].

A recent analysis of the 30 low-income countries with the most progress for primary health care services and outcomes in the last 30 years found that the top band of countries who now have comprehensive health systems had built these in a similar manner—starting with packages of care that were selective and increased in complexity over time [16]. Another key factor was an effective district health management system and willingness to adopt task-shifting, especially when building up the system.

Functional health systems are a prerequisite for comprehensive antenatal and childbirth care, which may serve as a platform for delivering most of the interventions discussed in the previous sections. It is possible that delivery and development research could contribute to delivering some of these interventions in a simpler way and at lower levels of care. Complex, facility-based interventions tend to have a higher level of inequity than simpler interventions that can be delivered closer to home. For example, there is low inequity for immunization and antenatal care, while higher disparities exist for skilled attendance coverage.

Issues related to scaling up are discussed in detail in the following pages within the context of four specific interventions: syphilis screening and treatment, emergency cesarean sections, newborn resuscitation and kangaroo mother care.

## Choosing delivery channels for cost-effective interventions

Successful scale-up requires delivering cost-effective interventions to those who need them most. Approaches for reaching high coverage are known as "delivery channels," "delivery strategies" or "means of distribution" in the literature [17], and should not be confused with the use of the term "delivery" as in childbirth.

Delivery channels are not restricted to contact with healthcare providers. They can also include mass media campaigns encouraging women to seek antenatal care early in pregnancy; food fortification such as micro-nutrients; marketing approaches such as bed nets; and school and workplace efforts to encourage birth spacing or delay the first birth. In terms of providers and facilities, those often involved in pregnancy and childbirth care include community health workers; traditional birth attendants; midwives or skilled attendants in the home; first-level health facilities; and health centers and hospitals [18]. Choosing the best delivery strategy will depend on the characteristics of local health systems, such as geographic accessibility to fixed health facilities and the proportion of pregnant women who attend antenatal care in such clinics. Regardless of the type of provider, appropriate attention to ensuring high quality care is essential; this requires thorough pre-service and in-service training, as well as regular supportive supervision [19,20].

The steps for scaling up interventions are highly context-specific. They must start with a situation analysis to identify the main causes of preterm births and stillbirths in the community. For example, the relative burden of malaria, syphilis or birth asphyxia in the population must be measured directly or estimated on the basis of information from comparable settings. Such data should be combined with the known efficacy of existing interventions, to estimate how many lives may be saved by scaling up each intervention in that particular setting. The next step is to assess the most appropriate delivery channels for reaching high and equitable coverage in the area. For example, assess the geographical accessibility and utilization patterns of primary, secondary and tertiary health facilities, the presence of relevant non-governmental organizations, the role of the private sector in delivering health care, and the availability of human resources for health care. Once interventions and delivery channels are selected, it is necessary to estimate how much the program will cost, and how it will be financed. Monitoring and evaluating programs to monitor success is a critical last step. In each of the above steps, consider which approaches will be most likely to improve equity as well as increase overall coverage.

This section stresses the need to reach high overall coverage in an equitable way, so that no population subgroup is neglected. Achieving equity is important because groups that are left behind are often those with the highest burden of morbidity and mortality, an unfair disparity in disease burden from the view of social justice [21,22].

## Scaling up interventions: concrete examples

Barriers to scaling up maternal, newborn and child health (MNCH) interventions include broad issues—such as human resources, financing of services, deployment of facilities, centralization, intersectoral policies, donor practices, and many others—that must be addressed by the health system as a whole. To help focus the discussion on preterm births and stillbirths, we now address more specific delivery issues. Based on the review in the previous article [1] on interventions, and using a Delphi process with substantial input from the Scientific Advisory Council and core investigators, four cost-effective interventions were selected:

1. Screening and Treatment of Syphilis

2. Emergency Obstetric Care (C-Sections)

3. Newborn Resuscitation

4. Kangaroo Mother Care (KMC)

Selection was based on their known cost-effectiveness and how they exemplify interventions delivered during pregnancy (syphilis screening), delivery (C-section and newborn resuscitation), and in early infancy (kangaroo mother care). They also cover the continuum of settings for providing healthcare, from communities to hospitals.

For each of the four interventions or packages, systematic literature reviews were carried out with a focus on delivery issues in LMICs. Further details on the literature search are provided in article 3 on interventions [1]. In addition, we broadened the review by searching for combinations of the terms "implementation', "scaling up", "scale up" and "coverage" with terms related to the outcomes under study ("preterm', "premature", "stillbirth', "antenatal care", "childbirth" and related terms). The search was conducted on PubMed and Google Scholar, and limited to publications and reports from low- and middle-income countries. The issues addressed below relate to coverage, equity, constraints and facilitating factors. The review also includes what is known from large-scale implementation studies.

### Antenatal care: screening and treatment of syphilis

Syphilis is common in LMICs, with prevalence among pregnant women varying widely: from less than 1% to 10% or higher [23]. An estimated two million pregnancies are affected every year; one in four of these ends in stillbirth or spontaneous abortion. Another 25% of these pregnancies result in newborns with a low birth weight or serious infection, both of which are associated with an increased risk of neonatal death [24].

Syphilis is presented as an example of a disease that can be effectively detected and treated by evidence-based antenatal care [25]. Major benefits for the mother and fetus include prevention of stillbirth and congenital syphilis. It is estimated that over 500,000 cases of congenital syphilis occur each year, and screening is a highly cost-effective antenatal intervention, even in low prevalence settings [26,27].

Other examples of diseases that may be detected and treated include conditions such as asymptomatic bacteriuria, HIV/AIDS and pregnancy-induced hypertension. The delivery challenges described below certainly apply to these other interventions as well.

#### Coverage and equity

When assessing equity of interventions preventing stillbirth or preterm birth, it is useful to consider three questions. The first is whether programs are designed with the specific aim of reducing inequities in access to effective interventions. Second, whether inequities in coverage were reduced as a consequence of the program.And third, whether inequities in morbidity or mortality decreased as a consequence of the program. Most of the information available refers to equity of coverage, and few if any studies have assessed equity in terms of morbidity or mortality.

Very limited information is available on the coverage of syphilis screening during antenatal care in LMICs, but syphilis screening cannot be greater than the coverage of antenatal care. Data from demographic and health surveys (DHS) conducted in 56 LMICs show that the global proportion of women with one or more antenatal visits is estimated at 76.1%, ranging from 47.4% in South Asia to 91.5% in Eastern Europe/Central Asia (Table 2). The more stringent indicator of three or more antenatal visits results in a lower global coverage of 63.2%, again with wide regional disparities.

**Table 2:** Percent of Births According to Antenatal and Delivery Care (in the Five Years Before a Recent DHS Survey)

	Number of Antenatal Visits to a Medically Trained Person		Delivery care		
		
Region	1 +	3+	Medically-Trained Person	Doctor	Health Facility
East Asia/ Pacific	75.9	62.6	60.7	21 .6	42.3
Eastern Europe/ Central Asia	91.5	75.7	94.9	72.7	91 .6
Latin America/ Caribbean	80.9	79.4	67.2	46.5	66.2
Middle East/ North Africa	64.0	50.7	60.5	37.3	54.7
South Asia	47.4	29.7	21.8	14.5	17.5
Sub-Saharan Africa	77.2	62.0	46.6	6.7	46.2
All regions	76.1	63.2	55.3	24.3	52.6

Source: DHS in 56 countries, Gwatkin DR *et al.*[22]

Studies suggest that many women attending antenatal care (ANC) in LMICs do not have a blood test [28-30]. Given that not all blood tests carried out during pregnancy will consist of syphilis screening, the estimated global coverage of syphilis screening must be even lower. A rough estimate provided by key informants from 17 countries in sub-Saharan Africa is that two in every five (38%) women among those attending ANC are screened for syphilis [28].

It is likely that inequalities in syphilis screening follow a similar pattern as reported for overall ANC. Table 3 presents the frequency of a pregnant woman attending three or more antenatal care visits with doctors, nurses or trained midwifes. The data are organized by region within five wealth groups. The table is based on DHS carried out in 56 LMICs in recent years. The table includes the percentage of births in the five years before the survey for which there were three or more antenatal visits to a medically trained health worker. Within every region, women in the poorest wealth quintile are particularly underserved. In South Asia, for example, only one in eight women have antenatal visits.

**Table 3:** Percent of Births for Which There Were Three or More Antenatal Visits to a Medically Trained Health Worker (in the Five Years Before a Recent DHS Survey)

	Percent of Women with 3 or more Antenatal Care Visits to Trained Health Workers According to Wealth Groups (Quintiles)				
		
Region	Poorest	2^nd^	3^rd^	4^th^	Wealthiest	All Groups
East Asia/ Pacific	47.3	58.6	62.9	68.2	83.3	62.6
Eastern Europe/ Central Asia	64.6	70.9	77.8	80.6	86.8	75.7
Latin America/ Caribbean	62.3	74.8	83.0	88.6	93.9	79.4
Middle East/ North Africa	31.8	40.8	49.5	60.2	74.1	50.7
South Asia	12.6	17.5	26.0	37.5	65.0	29.7
Sub-Saharan Africa	47.8	55.0	61.5	69.4	81.3	62.0
All regions	48.3	56.5	63.4	70.7	82.4	63.2

Source: DHS in 56 countries, Gwatkin DR* et al.*[22].

As for overall coverage of syphilis screening, very limited information is available in terms of social inequalities. In the 2006 Vietnam Multiple Indicator Survey (MICS), 75% of the women in the poorest wealth quintile had attended antenatal care (1+ visit), compared to 100% in the top quintile. Nevertheless, only 13% among the poorest had a blood test of any type during pregnancy, corresponding to one in six women attending ANC. Among the richest, 69% had a test, or seven in every ten [29]. This finding suggests that inequalities in blood tests in general are even more marked than inequalities in ANC.

Summarizing the available evidence, many mothers— particularly in South Asia—fail to receive antenatal care by trained providers. On the positive side, ANC contact rates are surprisingly high in sub-Saharan Africa. In all regions, the poor are less likely to receive antenatal care than those in higher wealth groups. Specific information on the coverage of syphilis screening is unavailable for most countries, but many—possibly most—women attending ANC fail to have any type of blood test.

#### Barriers and facilitating factors

According to the WHO, four pillars form the basis of national action plans to prevent congenital syphilis (Table 4) [24]. A recent review of how well five high-income and nine LMICs were performing in terms of these pillars [31] showed the majority of countries did not meet every element proposed in the WHO action plan. Political commitment varied across the 14 countries. Congenital syphilis elimination goals were rare but all had universal screening. Linkages to appropriate case management services were identified in 11 countries, although a national governing body was not generally evident.

**Table 4:** Pillars for National Action Plans to Prevent Congenital Syphilis

	Number of Countries Complying with Recommendation	
	
Pillar/Step	High-income (n=5)	LMIC (n=9)
**Ensure Sustained Political Commitment and Advocacy**		
Elimination goals set	1	2
Universal screening recommended	5	9
Commited government funding with little or no outside support	5	4
International/national partnerships	2	7
Linkages to appropriate case-management services (HIV/PMTCT or STI prevention programs)	4	7
**Increase Quality and Access to Maternal and Newborn Health Services**		
Where services are available:		
• Measures to ensure all pregnant women are screened and tested	4	4
• Increase access to care and decrease barriers	4	4
Where no services are available:		
• Partnerships with NGOs/community organizations to ensure maximum coverage	0	5
• Health promotion programs for congenital syphilis, STIs, reproductive health issues	3	0
**Screen and Treat Pregnant Women and their Partners**		
Diagnosis and treatment of pregnant women and partners	5	4
Point-of-care diagnostic testing	1	2
Single dose treatment for pregnant women	5	3
Measures to ensure women remain uninfected during pregnancy	4	5
**Establish Surveillance, Monitoring and Evaluation Systems**		
Establish national level baseline data and effective reporting for cases in pregnancy and congenital syphilis	5	5
Develop/strengthen systems for monitoring	5	5
Develop/strengthen systems for evaluation	4	1
Develop indicators/proxy measurements of congenital syphilis and effectiveness of intervention programs	1	1

Source: World Health Organization 2005, Hossain M *et al.*[24,31]

Efforts to increase and improve access to care were noted in eight countries with recommendations to ensure all pregnant women were screened and treated. LMICs had often formed international partnerships. Guidelines for the diagnosis and treatment of pregnant women and partners were lacking in most LMICs. Point-of-care diagnostic testing was very uncommon. Surveillance programs were active in 10 countries while comprehensive details on monitoring and evaluation components including proxy congenital syphilis indicators were unavailable for nearly all. These results reveal several major gaps, mainly in LMICs.

This policy study did not address health systems support issues, such as training staff in diagnosing and treating gestational syphilis, continuous provision of consumables (syringes, test kits, drugs, etc.), and supporting district management teams in terms of programming and supervision. These issues are also likely to be problematic in most LMICs [17]. In summary, virtually all the constraints to scaling up that are listed in Table 1 apply to syphilis screening.

#### Experience from large-scale programs

Literature and experts were sought to obtain real-life examples of large-scale evaluations of syphilis screening programs. Only a few case studies were located.

In Mozambique, a strong effort for scaling up antenatal syphilis screening in two provinces led to increased coverage from 5% in 1992 to 60-95% consistently since 1999. The authors report that key elements to effective antenatal syphilis screening include "adequate workforce, facilities, coherent systems of care, community involvement, donor management, advocacy, and leadership" [32]. The impact on preterm birth rates, stillbirths or perinatal mortality is not reported.

In Nairobi, data on trends in syphilis prevalence among pregnant women were related to the introduction of a strengthened, decentralized prevention and control program in government clinics, focused on the fight against AIDS and STDs in a combined approach. The program included staff training, providing test kits and drugs, supervision, monitoring and evaluation activities. From 1995 to 1997, syphilis prevalence was reduced from 7.3% to 3.8%. The authors attribute the decline to the program implemented, although the before-and-after design leaves margin for alternative explanations [33]. Impact was not reported on preterm birth or stillbirth.

Another before-and-after evaluation comes from a rural area in Haiti, where syphilis serology testing was decentralized from one hospital-based laboratory to 12 out of the 14 provincial health centers. This ensures rapid feedback of results to pregnant women with immediate treatment. This increased utilization rates, and decreased the incidence of congenital syphilis by 75%, relative to baseline levels, in the three years after the program was implemented. Impact was not reported on preterm birth or stillbirth [34].

An evaluation of national programs in Bolivia, Kenya, and South Africa showed early screening and treatment were affected by several constraints: (a) most women presented for their first antenatal clinic visit after 6 months of pregnancy; (b) it took up to 4 weeks to have the test results available; (c) no clinic had a system for tracking positive women who did not return for their results; (d) there were no guidelines for providers in Kenya and Bolivia; (e) in all countries, supplies, drugs, notification cards, and other consumables were often unavailable; (f) healthcare workers were unmotivated in Kenya; and (g) in South Africa and Kenya information on why blood had been collected was not provided. Many women, at their exit interview, stated they had never heard of syphilis nor had they been informed why blood was collected. The authors discuss several measures to improve the coverage and quality of screening and treatment [35].

The WHO review (Table 4) showed that lack of systems and tools for evaluation is one of the weakest pillars for congenital syphilis prevention. The scarcity of evaluative studies in the literature—made evident by the fact that only a few reports were available, and all of them lacking a comparison group—confirms this is indeed a major research gap. In particular, none of the evaluations reported on the impact of the interventions on preterm birth and stillbirth.

#### Research and implementation gaps

Based on the studies and evaluations reviewed above, we identified the following major research and implementation gaps that must be addressed:

• **Lack of visibility.** The fact that few countries have a syphilis eradication goal is a clear indication of the lack of political priority. Congenital syphilis is a preventable disease, screening is highly cost-effective, and the necessary diagnostic and treatment tools have been available for decades. Yet, globally, there are an estimated 500,000 annual fetal deaths from congenital syphilis, a figure similar to that from mother-to-child transmission of human immunodeficiency virus (HIV), which receives far greater attention in spite of being less cost-effective to prevent [26,36]. The prevention of congenital syphilis should be more of a global priority; international agencies and national programs should be committed to improving antenatal care (ANC) services including syphilis detection and prevention. The poor visibility of syphilis prevention is related to the broader issue of lack of visibility for stillbirths, a major public health problem (Table 1).

• **Lack of integration.** As a consequence of its poor visibility, it is not surprising that syphilis prevention is poorly integrated with other programs. In many LMICs there is a lack of clarity about whether antenatal, family planning, or programs on sexually transmitted diseases are responsible for syphilis screening in pregnant women [37]. The poor integration of these vertical programs hinders health care delivery at local levels, as does lack of coordination and conflicting agendas of donors – reflected in the contrast between the large availability of funds for preventing mother to child transmission of HIV (PMTCT) and the low investment in congenital syphilis prevention [36].

• **Need for point-of-care syphilis testing.** A major opportunity for expanding syphilis prevention resides in the observation that most mothers in LMICs attend antenatal care services at least once during pregnancy, with the notable exception of South Asia. Point-of-care, heat-stable rapid tests that require a single contact with health services are now available and have been tried in several LMICs, and yet the WHO review (Table 4) showed few countries have adopted them. Their simplicity and limited requirements for electricity and equipment suggest their use could improve the coverage of antenatal syphilis screening in developing countries [27,38-40]. Their wider adoption is still limited by the fact that point-of-care tests tend to cost more than traditional tests performed at a laboratory facility. However, this higher cost is largely offset by the fact there are no dropouts between testing and returning to receive the results [38]. Point-of-care testing represents an urgent implementation gap.

• **Need for research on oral treatments.** Gestational syphilis is traditionally treated with injectable benzathin benzylpenicillin on a single occasion. In light of the risks associated with injections in LMICs [41], adoption of oral treatment would reduce risks and possibly increase compliance. Single dose oral azythromycin has been tried but results have not been uniform [42].

• **Need for a vaccine.** The sequencing of the genome of T. pallidum ten years ago raised expectations regarding the development of a vaccine to prevent syphilis. However, progress has been limited. This has been plagued by the difficulty in cultivating the organism for microbiological study and the lack of identification of a reasonable antigen as a vaccine candidate. Greater funding might lead to breakthroughs in this area [43].

• **Need for research on missed opportunities among women who attend ANC.** This review showed that a substantial proportion of women who attend ANC are not screened for syphilis. Experts in the field raise the possibility that concern about HIV PMTCT has negatively affected prevention of congenital syphilis [36]. Audit and confidential inquiry programs and operational research are needed to understand the reasons for such failure.

• **Need for research on how to increase coverage through alternative delivery channels.** Operational research on how to overcome barriers to screening in poor populations—particularly in South Asia where ANC coverage is the lowest in the world—is essential for scaling up the prevention of congenital syphilis. The marked social inequities (described in Table 2) must be overcome through specific strategies aimed at providing ANC to the poor. In Mali, for example, the UNICEF-led Accelerated Child Survival and Development initiative used outreach to extend ANC to rural areas and led to a marked reduction in social inequalities in coverage, although this study did not report specifically on syphilis screening [44]. Promoting early attendance to ANC is also essential for maximizing the benefits of syphilis screening. In areas where access to health facilities is limited, pilot studies should be carried out with rapid point-of-care testing and oral treatment by community healthcare workers or traditional birth attendants. 

• **Cost-effectiveness of alternative approaches to antenatal screening.** In areas with low coverage of health services, cost-effectiveness studies are needed to examine alternative control strategies. These include mass and targeted treatment in high-prevalence populations [42], as was done in Uganda [45] and proposed for Kenya [46].

### Delivery care: emergency obstetric care (cesarean sections)

Intrapartum deaths account for 30% of all stillbirths— over one million deaths a year. Cesarean sections (C-sections) are essential for preventing fetal death due to obstructed labor, cord prolapse, breech presentation, and other conditions, as well as maternal deaths [18]. C-sections are one of the key elements of comprehensive emergency obstetric care (CEmOC). Issues related to making C-sections available to women in need are discussed as an example of the broader need for CEmOC.

#### Coverage and equity

Unlike interventions needed by all pregnant women, C-sections are estimated to be required in 5-15% of all births [47,48]. Estimates from LMICs suggest a C-section rate of 12%, with regional rates ranging from 3-26%. Rates seem to be increasing in most countries except in sub- Saharan Africa, where little, if any, change has occurred [49]. Apparently, adequate national C-section rates often hide important social differentials. Data from 42 LMICs show that cesarean rates lower than 1% are found among the poorest 20% of the population in 20 countries, and in only five countries the rate among the poorest exceeded 5%. Fourteen countries—Haiti, Nepal and 12 countries from Sub-Saharan Africa—had national rates of less than 2.0%. At the other extreme are seven countries, mostly in Latin America, where C-sections are far in excess of the suggested maximum threshold of 15% for at least 40% of the population [50]. Table 5 summarizes these results, showing the unweighted average C-section rates in sub-Saharan Africa (27 countries), Asia (six countries) and in Latin America and the Caribbean (nine countries). The table shows the percentage of births in the five years before the survey in which a cesarean section was performed.

**Table 5:** Percent of Births in Which a Cesarean Section was Performed (in the Five Years Before a Recent DHS Survey)

	Percent of C-sections According to Wealth Groups (Quintiles)					
		
Region	Poorest	2^nd^	3^rd^	4^th^	Wealthiest	All Groups
Asia	1.5	2.2	3.6	6.9	15.6	5.3
Latin America/Caribbean	6.7	13.1	19.0	25.7	38.3	18.4
Sub-Saharan Africa	1.4	1.9	2.4	3.3	7.8	3.1
All countries	2.5	4.3	6.2	8.6	1 5.4	6.7

Source: DHSs in 42 LMICs, Ronsmans C et al. [50]

Inequalities in access to C-sections reflect broader inequities in delivery care. Table 6 shows that in every region—except for the former Soviet bloc countries in Europe and Asia— women in the highest quintile of the socioeconomic distribution are at least twice as likely to have a skilled attendant at childbirth as those in the poorest quintile.

**Table 6:** Percent of Births That Were Attended by a Medically-Trained Worker (in the Five Years Before a Recent DHS Survey)

	Percent of C-sections According to Wealth Groups (Quintiles)					
		
Region	Poorest	2^nd^	3^rd^	4^th^	Wealthiest	All Groups
East Asia/ Pacific	34.4	53.7	65.9	75.8	91.7	60.7
Eastern Europe/Central Asia	88.4	94.6	96.7	98.2	99.2	94.9
Latin America/Caribbean	45.4	59.0	71.1	83.9	93.6	67.2
Middle East/ North Africa	39.7	51.4	61 .3	72.2	84.6	60.5
South Asia	7.0	10.4	17.0	28.3	56.0	21.8
Sub-Saharan Africa	25.6	34.2	42.9	59.3	82.5	46.6
All regionsD	35.8	45.5	54.3	67.3	85.0	55.3

Source: DHSs in 56 LMICs, Gwatkin DR et al. [22]

Socioeconomic inequities in skilled delivery care show an important overlap with urban and rural disparities, as the poor are often concentrated in rural areas. A recent review concluded that "progress in professionalization of childbirth… is held back by a marked stagnation in rural areas, mainly in sub-Saharan Africa and South and Southeast Asia where rural populations still constitute a large proportion of total populations" [9]. Typically, rural C-section rates are about a third of urban rates, even in countries where urban rates are below the minimally recommended [9].

#### Barriers and facilitating factors

There is a wealth of literature on C-sections and other aspects of emergency obstetric care. The vast majority of authors address issues of maternal mortality, a few deal with newborn survival, but less concentrate on stillbirth. Even the heated discussion on the ideal proportion of C-sections is largely centered on maternal indications, rather than indications related to fetal or newborn health [51-53]. Given that lack of emergency obstetric care is a major cause of intrapartum deaths, this is yet another example of the invisibility of stillbirths, even within the medical literature.

In this section we will refer to four service-delivery models for childbirth proposed by Koblinsky and Campbell [8]:

• Model 1 - home delivery attended by a non-professional

• Model 2 - home delivery attended by a skilled birth attendant

• Model 3 - labor supported by a skilled attendant working in a health facility providing basic emergency obstetric care (BEmOC), including parenteral drugs (antibiotics, oxytocic drugs and anticonvulsants), manual removal of retained placenta, removal of retained products of conception, and assisted vaginal delivery

• Model 4 - comprehensive emergency obstetric care or CEmOC, entails hospital delivery for all women by skilled attendants with the ability to perform C-sections and blood transfusions

Models 1 and 2 refer complicated cases to facilities [47,54].

Several high-profile documents [18,55,56] strongly support models 3 and 4, opposing model 1 home deliveries by traditional birth attendants. This view is not universally shared [57]. Other authors are rightfully concerned with a one-size-fits-all strategy, pointing out that "intrapartum care based in health centers is appropriate for all as a longer-term strategy, but might not be the best option for reducing maternal mortality in all contexts in the shorter term" [58].

In many LMICs, where model 1 prevails, women with complicated labor often face four major delays: recognizing complications; deciding to seek care; reaching a health facility due to lack of transportation or resources; and, lastly, receiving appropriate care at the facility [59-61]. In these settings, the shortfall of professional care is a key constraint. The World Health Report 2005 estimated that nearly three times the current number of professionals—about 700,000 more—are needed for full coverage of women during childbirth by 2030 [56].

In settings where services are available but underused, there may be important demand-side barriers, for example reluctance or inability of mothers to use services. These include economic barriers (costs of services), geographic barriers (need for transportation and associated costs) and cultural barriers (lack of decision making power by women) [9]. Poor perceived quality of services may also contribute to low utilization in settings where access is not an issue.

A promising approach to reduce demand-side barriers, specifically out-of-pocket expenses resulting from facility deliveries—is that of conditional cash transfers. [62] Most positive examples, however, come from Latin America, and it is debatable whether similar impact would be achieved in other settings. In Nepal, a scheme of cash payments to pregnant women from districts with very low utilization of hospitals for delivery has resulted in increases in hospital delivery in areas where women's groups had a strong presence, but there was no measureable impacts on C-section rates [63]. Given the importance of out-of-pocket costs resulting from facility delivery, further evaluations are needed of the potential benefits of conditional cash transfers and similar schemes.

In most countries, C-sections are solely carried out by medical doctors. This represents a major barrier because doctors are estimated to carry out only one in four deliveries in LMICs, ranging from 6.7% in sub-Saharan Africa to 73% in Eastern Europe and Central Asia (Table 2). In the poorest quintile of the population, these proportions are as low as 2.5% in sub-Saharan Africa and 3.4% in South Asia [22]. Many high-mortality countries suffer from critical health worker shortages. Sub-Saharan Africa accounts for about half of all under-five deaths and has only 3% of the global health workforce [64,65].

#### Review of large-scale experiences

As above, the review of national experiences is focused on evaluations with maternal mortality as the endpoint, rather than stillbirths. Because C-sections are essential to preventing both maternal mortality and stillbirths, the maternal literature will be relied upon. In view of the large amount of literature on experiences with different degrees of success in ensuring access to CEmOC in LMICs, three major reviews were chosen and summarized.

According to an ample review by WHO:

*"[C]ountries that have successfully managed to make motherhood safer have three things in common. First, policy-makers and managers were informed: they were aware that they had a problem, knew that it could be tackled, and decided to act upon that information. Second, they chose a common-sense strategy that proved to be the right one: not just antenatal care, but also professional care at and after childbirth for all mothers, by skilled midwives, nurse-midwives or doctors, backed up by hospital care. Third, they made sure that access to these services—financial and geographical—would be guaranteed for the entire population" *[66,67].

These three steps are well in line with the discussion of constraints (Table 1) as well as with the previous section on inequalities.

Sri Lanka and Malaysia are textbook examples of countries that reduced maternal mortality by providing skilled birth attendants and supportive facilities. In these countries, maternal mortality ratios were halved every 7-10 years, over successive time periods. Removing financial barriers to care with marked improvements in equity in access were key elements of their success [68].

Koblinsky et al [69] reviewed the progress in Malaysia, Sri Lanka and rural China (where maternal mortality was also substantially reduced), identifying six key elements: high availability of skilled birth attendants located near the home (especially where home births are traditional); high availability of birthing facilities; free or reduced costs for services and transportation; strong government policies for maternal heath; formalized referral links among facilities and providers (including community providers such as traditional birth attendants (TBAs); and providers accountable to the public for their performance. These authors expanded their review to cover recent successful experiences in Bolivia, Egypt, Honduras, Indonesia, China and Zimbabwe. They found the first three elements—close-to-home attendants, high facility availability, and removal of financial barriers— were also present in these countries, but the other three elements were not. This led to the conclusion that although all six elements are important, progress may be observed when not all are achieved.

#### Research and implementation gaps

A full gap analysis of issues related to ensuring universal access to emergency C-sections in LMICs, a key element in reducing intrapartum stillbirth, is well beyond the scope of this report. Instead, we concentrate on a few high-priority issues: human resources, access, referral, and costs.

• **Need to deploy human resources: task shifting.** The devolution of selected clinical responsibilities to health worker cadres with shorter training is increasingly seen as an option to address health worker shortages [70]. Freedman uses the example of obstetric anesthesia to show that relying on nurses, compared to anesthesiologists, to deliver this procedure would likely increase case-fatality. However, the increase in population coverage would more than offset the added mortality [71]. Experience from Mozambique suggests that medical assistants trained in surgery ("surgical technicians") can perform C-sections with results that are virtually identical to those obtained by obstetricians [72,73]. This approach allowed the provision of C-sections in areas of the country where doctors were not available [74]. Similar results were reported from the Democratic Republic of Congo [75]. Rapid turnover of highly skilled staff is a major problem in LMICs. In Mozambique surgical technicians, as opposed to doctors, tended to remain in district hospitals where they were deployed [76]. An important policy constraint regarding task shifting is resistance by professional medical organizations to delegate specific activities to other cadres (see, for example www.wma.net/e/press/pdf/task_shifting_050308.pdf). Further operational research on task shifting is urgently needed. Research gaps include larger studies of the effectiveness of non-doctors performing C-sections, with sufficient sample sizes. Such studies should not only compare the success rates of doctors with non-doctors, but also take into account that a lower success rate may be acceptable if coverage is expanded substantially. Studies must also address how to overcome the resistance of professional organizations to training surgical technicians and determine the best strategies for reducing staff turnover.

• **Need to improve referral for complicated deliveries.** In high- and middle-income countries, the vast majority of deliveries take place in a hospital or similar facility that is able to provide CEmOC for complicated labor (Model 4). This is unlikely to be the case in the near future for most low-income countries where deliveries occur at home or in small health facilities (Models 1-3). The issue, therefore, is how to ensure the timely referral of mothers who need a C-section to a facility providing CEmOC. The "risk approach," popular in the 1970s and 1980s, proposed that women needing to deliver in a facility could be identified during antenatal care and referred to a hospital close to the date of delivery [77]. Global experience, however, showed that many, if not most mothers who will need a C-section cannot be identified successfully before labor starts [66]. This recognition led to several complementary approaches:

• **Birth preparedness.** This approach has been promoted in several African countries, and consists of preparing the mother, family and community for home deliveries and potential complications. It includes identifying the place of delivery, acquisition of sterile materials (blade, soap, cord ties, clean linen) and planning for referral if needed – including setting aside money and arranging transportation to a facility [78]. In theory, birth preparedness makes sense by addressing delays, but it has not been sufficiently evaluated, [79-82] as noted in article 3 on interventions. Rigorous evaluations are needed to assess its effect under real-life conditions.

• **Maternity waiting homes.** These are places, typically near a hospital, where pregnant women can stay near the time of delivery, thus precluding the need for travel in case of complications. Several case-studies in Honduras, Cuba, Ethiopia and Zimbabwe suggest that waiting homes increase access to CEmOC and may reduce intrapartum fetal deaths [69]. Studies on cultural and economic barriers to using maternity waiting homes (e.g., the woman needing to be away from home for a potentially long period) are needed to predict costs and compliance rates.

• **Timely referral by attendant.** Regardless of who is attending the birth in a non-CEmOC setting, delays in referral must be avoided when the need arises. The partogram is a key tool for early recognition of complications in labor and allows for prompt referral [83,84]. This tool, used to assess the progression of labor and delivery, documents cervical dilation and fetal heart rate over time and alerts the attendant to slow or abnormal progress. In theory, the partogram should help refer women who need a C-section from delivery Models 1-3 to a Model 4 facility. However, evaluations of the parto- gram show a reduction, not an increase in C-section rates [83,85]. All studies were conducted in hospital settings. In spite of strong recommendations supporting use of the partogram in all settings, surveys in different LMICs show that although most have knowledge of the partogram, only a small proportion of skilled birth attendants effectively use this tool and even fewer actually monitor the fetal heart effectively [86-88]. Research gaps include the impact of the partogram on referral, and ultimately on stillbirth outcomes, when used for labor taking place at home or in small facilities; how use of the partogram can be scaled up among skilled attendants; and ways to improve simple, robust technology for fetal heart rate monitoring.

• **Need to reduce financial barriers to CEmOC.** Out-of-pocket payments are the principal means of financing health care including child birth in most of Africa and Asia [89,90]. Marked disparities in coverage exist between wealth groups for skilled delivery in the public sector in most LMICs, and to an even greater extent in the private sector [22,91]. Reviews of success stories in scaling up skilled delivery care unanimously concluded that removal of financial barriers were a key element for reducing mortality [66-69]. The introduction of user fees in government facilities in many countries in the late 1980s and early 1990s exacerbated inequalities, [92] and mechanisms for protecting the poor from user fees through exemption schemes have failed in several countries [92-94]. Once user fees are implemented, however, their sudden removal without proper alternatives for financing services can also be disastrous, as shown in South Africa and Uganda where staff salaries were negatively affected with a marked impact on their morale [95]. A key implementation gap is to reduce or abolish user fees in the public sector, while increasing and effectively disbursing public funding for these services to maintain adequate quality. Innovative approaches— including conditional cash transfers—are needed to reduce other expenses incurred by the poor in obtaining services, such as transportation costs and loss of income while receiving care [96].

### Newborn care: resuscitation

Five to 10% of all newborns will need some assistance to begin breathing [97,98] and about 1% will require extensive resuscitation [97]. Use of a bag and mask (ambu bag) or mouth-to-mask (tube and mask) device at home or in a local health facility will save four out of every five babies who need resuscitation; more complex procedures, such as oxygen and/or endotracheal intubation, are required only for a minority of babies who do not breathe at birth [59,99]. Because the need for resuscitation cannot be predicted prior to delivery, all birth attendants should be proficient in this practice and have the appropriate equipment available both in term and preterm births [54].

#### Coverage and equity

Resuscitation is only required by a small and variable proportion of newborns, and has only recently started to receive wide attention. As a consequence, data on coverage of this intervention—such as the proportion of children who were ventilated among those who did not breathe at birth—are hard to obtain. One may assume, however, that few if any infants delivered at home in LMICs are currently resuscitated. For infants delivered in facilities, surveys in several LMICs suggest that typically half or fewer of all skilled attendants—doctors and midwives—working in institutions have resuscitation skills [78,86,100]. Because about 55% of births in LMICs are attended by a skilled provider (Table 6) one may conclude that a quarter or so of all babies suffering from asphyxia receive resuscitation. Because socioeconomic inequalities in skilled attendance are vast, inequities in resuscitation coverage must also be large.

#### Barriers and facilitating factors

A thorough review concluded that "the main barriers to effective resuscitation are lack of competent staff and lack of simple equipment" [59]. Unlike C-sections, which require highly skilled staff and complex equipment, neonatal resuscitation may be carried out by any type of provider, given minimal training and using simple equipment. Thus, barriers precluding access to health facilities by the poorest mothers do not necessarily deprive their infants from being resuscitated if needed.

There is little question that skilled health workers— doctors, nurses or midwives—are the first choice for providing delivery care, including resuscitation. However, universal coverage with skilled attendants at childbirth is still a far cry for many poor countries (Table 2) where a majority of deliveries are carried out by TBAs. Fortunately, TBAs may be successfully trained in resuscitation [101-103]. Ideally, training requires the use of a baby "dummy" equipped with inflating bags and blow-off valves, to teach the attendant how to supply the correct amount of air [59]. As for any TBA-training intervention, an important barrier is how to identify these providers and attract them to training courses. This is compounded by the fact that many TBAs only deliver a few babies a year, which seriously affects the cost-effectiveness of training by requiring a much larger number of trainers to reach high coverage [9].

Adequate equipment must be available to all providers who are trained. Inflating bags are available for about US $6 but mass production can reduce costs [59]. Distribution of equipment at low or no cost may serve as an incentive and should be coupled with training. After equipment is distributed, measures must be in place to replace faulty or lost pieces, as well as to provide new birth attendants with bags and masks.

#### Review of large-scale experiences

There are several good examples of how training staff in neonatal resuscitation and providing the required equipment may help prevent neonatal deaths. Most reports, however, refer to relatively small-scale programs such as in a few hospitals. Some examples are discussed below.

A program in Zhuhai City, China used evidence-based guidelines to establish a neonatal resuscitation program. Training materials were developed and certification provided for providers who attended the course. Over a two-year period, the early neonatal mortality rate dropped significantly from 9.9 to 3.4 per 1,000 live births [104].

The impact of a neonatal resuscitation program on the incidence, management and outcome of birth asphyxia was evaluated in 14 teaching hospitals in India. Two faculty members from each institution attended a neonatal resuscitation certification course and afterwards trained staff in their respective hospitals. The program led to increased awareness by staff and more rational resuscitation practices; the authors also report a significant (P<0.01) decline in asphyxia-related deaths, but the magnitude of the reduction is not described [105].

We located two reports on programs from LMICs regarding national programs aimed at scaling up neonatal resuscitation.

In India, the Neonatal Resuscitation Programme was launched in the 1990s with support by the American Academy of Pediatrics and American Heart Association. Its initial goals included training of trainers and provision of the necessary equipment. A national faculty of 150 pediatricians and nurses was trained in various regions of the country, who then trained 12,000 providers in several states over the following 2 years. Resuscitation was also introduced in pre-service training of medical and nursing students in several institutions [106].

An evaluation of the Malaysian Neonatal Resuscitation Program addressed the issue of "cascade-training" as part of scaling up. The original 37 core instructors of the national program were followed-up for two years; 35 carried out training courses in their respective home states, leading to a further 513 new instructors and 2,256 providers being trained subsequently in all 13 states. However, 61% of the 550 instructors were inactive trainers, having each trained fewer than four health workers in a year. An initial evaluation highlighted the relative inefficiency of training, with over half of the trainers failing to effectively disseminate the knowledge acquired [107]. More recently, a before-and-after impact evaluation (1996 to 2004) suggested there was no impact on stillbirth rates (4.3 per thousand in 1996 and 4.1 per thousand in 2004), but neonatal mortality declined from 9.1 per thousand in 1996 to 6.8 per thousand in 2004 [108].

#### Research and implementation gaps

Neonatal resuscitation is a low-cost, low-tech intervention that can save hundreds of thousands of newborns every year. Yet, fewer than one in every four babies who need resuscitation receive it. Which factors preclude it from being done more often? A discussion of key implementation and research gaps follows. 

• **Need to scale up training with quality.** The successful experiences in scaling up training in resuscitation, as in India and Malaysia, must be further expanded and exported to other countries. Key research questions include how to expand training coverage rapidly without losing quality? How to ensure instructors continue to train other providers after returning to their home bases? How to include resuscitation skills in pre-service training of doctors, nurses and midwives? How to organize refresher training so that skills are retained over long periods of time?

• **Need to involve private providers.** Experience in scaling up training in resuscitation is largely limited to government health workers employed in public hospitals and clinics. A major implementation and research gap is how to identify, attract and train private providers, from doctors to TBAs, and how to ensure they retain and apply their skills after being trained. Potential cultural barriers to use of resuscitation techniques and equipment—for example, cultural attitudes towards a newborn that appears to have died—must also be investigated.

• **Need to make resuscitation equipment widely available.** Although the equipment for resuscitation is inexpensive and simple to manufacture, there are important challenges in making hundreds of thousands of units available to the world's providers. A major implementation gap is how to produce and distribute equipment at such a massive scale.

• **Need for audit systems.** Perinatal audit is important for both neonatal resuscitation and for identifying potential failures of the health system in preventing deaths associated with conditions other than birth asphyxia—such as neonatal syphilis or obstructed labor—as well as missed opportunities in preventive interventions such as antenatal steroids for preterm birth. Many high-income countries have such systems in place. In South Africa, 30% of all births are covered by a perinatal problem identification program [109] but such systems are rare in other low- or middle-income countries. Implementation research is needed to disseminate audit systems widely.

### Kangaroo Mother Care (KMC)

As discussed in the interventions article in this report [1], KMC is a cost-effective approach for reducing neonatal morbidity and mortality. Virtually all of the global experience with this method relates to hospital settings in LMICs. There are three main components of hospital-based KMC: kangaroo positioning of the infant, allowing skin-to-skin contact in a vertical or semi-vertical position; exclusive breastfeeding; and early discharge from the hospital with appropriate follow-up practices [110].

#### Coverage and equity

With the possible exception of some indigenous populations, skin-to-skin contact between mother and infant seems to be very rare. For example, in the control group in the community-based Bangladesh trial [111], less than 1% of the newborns had skin-to-skin contact with the mother. Similarly low rates were found at baseline in an Indian trial [82].

KMC is being implemented on a large scale in a few countries, including Brazil and South Africa. In Brazil, 326 (7.3%) hospital units out of a total 4,490 in the country have joined a national program that promotes KMC for children under 2,000 g at birth (see article http://portal.saude.gov.br/portal/saude/visualizar_texto. cfm?idtxt=30076& janela=1). In South Africa over half of all hospitals have some form of KMC practice (R. Pattinson, personal communication). Scale-up is also underway at a more limited scale in Malawi and some hospitals in developed countries practice either full KMC or parts of it (e.g., skin-to-skin contact and exclusive breastfeeding).

No information on equity of KMC coverage is currently available, but given that it is provided primarily in hospital settings, children who are delivered at home (Tables 2 and 6) will not benefit until facility-based KMC is brought down to the lower level of facilities and linked to communities. Further development research for community-based KMC is also required.

#### Barriers and facilitating factors

A KMC center in Bogota, Colombia, trained 44 healthcare teams from 25 LMICs [110]. Follow-up with the trainees after returning to their countries of origin identified important barriers to implementation. These barriers include the perception that KMC is the "poor man's alternative" to more sophisticated care; increased work for hospital staff; cultural objections to direct and continuous contact between a naked baby and its mother, exposure of the mother's body to medical staff; objections against exclusive breastfeeding and perception of formula feeding as more modern and sophisticated; resistence of hospital staff to early discharge practices; and lack of policies and resources for follow-up. Although most of these perceived barriers can be overcome, they represent real obstacles that must be faced when scaling up KMC.

In South Africa, a typology of progress toward scaling up KMC at the hospital level identified six implementation phases: increasing awareness, adopting the concept, mobilizing resources, delivering evidence of practice, including evidence of routine, integration, and sustainable practice [112]. This scale was used to compare different implementation strategies, including provision of a standard implementation package with and without visits from a facilitator, [113] and on-site versus off-site facilitation [114]. For all approaches, most hospitals showed evidence of practice, implying that the strategy can be implemented successfully. Ongoing, onsite facilitation was associated with stronger implementation than mere provision of the packaged materials.

Experience with KMC implementation at the community level is much more restricted than hospital-level initiatives [115]. The Bangladesh RCT was inconclusive in terms of impact; its authors recommend that "additional experimental research. is needed to determine whether community KMC benefits newborn and infant survival" [111]. The study also showed that in spite of strong promotion only 24% of the mothers in the intervention group complied with skin-to-skin contact for seven hours or longer in the first two days after delivery; by the second week of life, average skin contact was 1.2 hours per day. Low compliance suggests the presence of important obstacles to implementation, at least in this society. One such barrier was that existing community nutrition workers were unable to sustain the intervention, and additional health workers had to be recruited [111]. There were also concerns that very preterm neonates may be kept at home too long instead of being taken to facilities. An Indian trial [82] reported high rates of compliance with initial skin-to-skin contact (over 80% in two intervention groups, compared to 10% in the control group) but no information is provided on how many hours a day contact was maintained, nor on how long the effect remained. There seem to be barriers for scaling up KMC, particularly in the set-up phase when doctors and nursers need to change practices and allow mothers access to neonates 24 hours a day. In many cases scale-up has been led by influential champions to get the process of change started. Succesful implementation in many hospital settings suggests that such barriers can be overcome. Additional research is needed to identify and remove them and to examine cultural barriers for families, especially in South Asia.

#### Review of large-scale experiences

Only two countries provide information on the likely health impact of KMC. In South Africa, a study assessed the impact of the introduction of KMC in 40 hospitals participating in a perinatal network. Neonatal mortality for infants born with 1,000 to 1,999 g was 19% lower in hospitals with KMC than in those without it (88 and 71 deaths per thousand, respectively). In 11 hospitals with time series information, KMC introduction was associated with a 38% reduction (from 88 to 61 per thousand) in neonatal mortality (RR 0.62; 95% CI 0.53-0.73) [116].

In Brazil, an ecological study was performed in the country's 27 state capitals including all 110 high-risk maternity units. Information on the implementation of KMC and availability of technology was obtained by postal questionnaires from 97 units. Late neonatal mortality (7-27 days) was inversely associated with the strength of KMC implementation (R=-0.43; p<0.01) after adjustment for the technology score of the maternity unit and region of the country [117].

There is limited evidence on the large-scale effectiveness of KMC under real-life implementation conditions, but the two observational studies reviewed above suggest a beneficial impact on neonatal mortality. The impact on neonatal mortality in HIC settings may differ from what is observed in LMICs and, therefore, should be studied further to determine the value of the KMC approach.

#### Research and implementation gaps

KMC is an effective intervention that can save newborn lives at low cost in hospital settings. Yet, 30 years after it was originally proposed, it is still received with skepticism by many health workers and policymakers. Often KMC is restricted to tertiary hospitals despite the potential for practice at lower level facilities. The main research gaps include:

• **Feasibility, effectiveness and safety of community- based KMC.** The only study on the impact of community KMC was inconclusive [111]. Well-designed community trials are urgently needed to establish its cost-effectiveness. Such trials must properly assess birth weight and/or gestatational age of the children enrolled, as well as identify cultural and health system barriers to implementation and investigate how these may be overcome.

• **Need to overcome barriers to implementation in hospital settings.** Operational research is needed to understand and overcome barriers by hospital staff, including health workers and managers, particularly related to the perception that KMC is not an effective intervention, or that it constitutes "medicine for the poor." Research is also needed on different approaches to training staff on KMC, including implementation of early discharge with adequate follow-up support and counter-reference to first-level health facilities.

• **Need to bring KMC closer to the population.** In most countries KMC implementation started at teaching or other tertiary hospitals. Operational research is needed on how to expand the KMC approach to district hospitals and even maternity units as is currently being tried in Malawi and Tanzania.

## Discontinuing ineffective interventions

An important cross-cutting delivery issue is how to discontinue interventions that are either ineffective, harmful or both. The literature on antenatal and delivery care is full of examples of such interventions. Extremes include deeply ingrained lay practices carried out by TBAs on babies who need resuscitation, such as slapping, blowing on, or pouring cold water on the baby; holding the baby upside down or giving injections [59].

The other end of the technology continuum includes overusing C-sections, episiotomies, tocolytics, and oxytocics in the early stages of labor, particularly in middle- and high-income settings [118]. Many of the procedures normally included in antenatal and delivery care have also been challenged due to potential harm and lack of evidence of benefit [119]. Whereas most authors address this issue from the providers' side, there is also consumer demand for medicalized care [120].

As a consequence, a major research gap to fill is identifying effective strategies for discouraging providers from carrying out ineffective and harmful procedures, and discouraging mothers and their families from demanding them. There is also a pressing need for realtime monitoring of the frequency of obstetric and newborn care interventions along with periodic evaluation studies regarding what proportion of these procedures are actually justified by medical indications.

## Placing preterm birth and stillbirth interventions in the broader maternal, newborn and child health context

Few health interventions affect a single outcome. There is considerable overlap between interventions targeted to prevent preterm birth and stillbirth and those that are also cost-effective for other maternal, newborn and child (MNC) conditions. Table 7 illustrates this overlap. The top left cell includes how to proceed with interventions this review identified as effective in reducing preterm birth and stillbirth and are known to be effective in reducing the morbidity and/or mortality of mothers, newborns and children. One such example is screening and treatment of syphilis. These interventions should obviously continue to be promoted, and findings on their ability to prevent preterm birth and stillbirth should be used to advocate for rapid scale-up.

**Table 7:** How Preterm Birth and Stillbirth Interventions Fit In Broader Maternal, Newborn and Child Health Context

	Cost-Effective Against Preterm Birth, Stillbirth, or both?	
	
Cost-Effective Against Maternal, Newborn and Child Deaths?	Yes	No
**Yes**	• Continue to promote • Use the evidence on stillbirth/preterm birth for further advocacy	• Continue to Promote
**No**	• Advocate for implementation • • Promote operational research for scaling up • Promote further research on the overall impact against MNC deaths	• If the intervention is widely used, advocate for discontinuing implementation

The top right cell lists how to proceed with interventions that, although have a proven effect on one or more MNC conditions, do not seem to affect preterm birth or stillbirth. An example is iron supplementation during pregnancy. These interventions also deserve continued promotion, but their scale-up is unlikely to reduce preterm birth or stillbirth. It should be noted that these interventions were not systematically reviewed in the present document.

Of particular interest to this review is the bottom left cell. Interventions in this category were identified as effective in reducing either preterm birth or stillbirth, or in improving the survival of preterm newborns, but do not seem to affect MNC morbidity or mortality through other pathways. An example is the use of antenatal steroids for preterm labor. It is necessary to advocate for the inclusion of these interventions in MNC packages. At the same time, further research is necessary to estimate their possible impact on other MNC indicators.

Finally, interventions in the bottom right corner are ineffective against preterm births, stillbirths, and other MNC conditions. An example is routine episiotomy. These interventions, if already implemented, should be discontinued.

## Conclusion

There are few documented success stories of scaling up interventions against preterm births and stillbirths. This is confirmed by a recent review of global progress in disease control. Levine et al. amassed 20 successful experiences from LMICs, of which only the prevention of neural tube defects in Chile was relevant to the reduction of stillbirths, and none to preterm births [121].

The four interventions reviewed in detail in this article constitute only a few cost-effective, proven approaches to reduce the burden of disease associated with preterm births and stillbirths. Several other interventions, described in article 3 of this report [1], are also ready for scale-up. The implementation and research barriers described here also apply to most if not all of these proven interventions. These research and implementation gaps must be urgently filled in order to result in high and equitable intervention coverage, thus preventing MNC morbidity and mortality.

This section proposed several research and implementation gaps that require attention. Research gaps identified in this article and in article 3 have been merged and are undergoing a standardized procedure for prioritization – the Child Health and Nutrition Research Initiative (CHNRI) method, [122]—to provide guidance to governments and funding agencies on which interventions deserve the greatest attention. These results are expected to be published later in 2010.

The next article in this report is a qualitative analysis of advocacy challenges and opportunities to improve visibility, policies and investments for research and implementation [7].

## Authors' contributions

CV wrote the article. CR helped conceive of this article as part of a global report on preterm birth and stillbirth, and participated in its design and coordination.

## Competing interests

The authors declare that they have no competing interests.
